# Methodology for Static Pressure Measurement Under Confined Spatial Conditions in the Low-Pressure Range

**DOI:** 10.3390/s26082354

**Published:** 2026-04-10

**Authors:** Pavla Šabacká, Jiří Maxa, Michal Bílek, Robert Bayer, Tomáš Binar, Petr Bača, Vojtěch Hlavička, Jiří Čupera, Jiří Votava, Vojtěch Kumbár, Lenka Dobšáková

**Affiliations:** 1 Faculty of Electrical Engineering and Communication, Brno University of Technology, Technická 10, 616 00 Brno, Czech Republic; 2 Institute of Scientific Instruments of the CAS, Královopolská 147, 612 64 Brno, Czech Republic; 3 Faculty of AgriSciences, Mendel University in Brno, Zemědělská 1665/1, 613 00 Brno, Czech Republic; 4 Faculty of Military Technology, University of Defence, Kounicova 65, 662 10 Brno, Czech Republic

**Keywords:** Ansys Fluent, aperture, CFD, differentially pumped chamber, ESEM, low-pressure, nozzle, Pitot sensors

## Abstract

This paper presents a methodology enabling the use of a Pitot probe for static pressure measurement in supersonic flow under severely confined spatial conditions where standard design guidelines cannot be satisfied. In particular, the recommended placement of a static pressure tapping at a distance of 10–20 tube diameters is not feasible; the proposed approach allows for the tapping to be located immediately downstream of the static tube cone. The methodology combines theoretical analysis, experimental measurements, and Computational Fluid Dynamics (CFD) simulations. Experiments were performed using appropriately selected pressure sensors, while detailed simulations in Ansys Fluent (Ansys 2024 R2) included both a high-fidelity probe model and free-stream flow analysis. By comparing experimental and numerical results, a correction coefficient was established based on the free-stream dynamic pressure obtained from CFD. This enables the accurate estimation of static pressure even in non-ideal probe configurations. The measurement error did not exceed 20%, while in most cases, very good agreement between experimental and CFD results was achieved. The main contribution of this paper is the validated methodology, which extends the applicability of Pitot probes to geometrically constrained environments where conventional static pressure measurement techniques cannot be implemented.

## 1. Introduction

The measurement of static pressure in small volumes, particularly in microfluidic systems and confined microenvironments, represents an important research area at the intersection of microelectromechanical systems (MEMS), optical methods, and experimental fluid mechanics. Static pressure is defined as a scalar quantity acting uniformly in all directions at a given point in a fluid, and its accurate measurement at the microscale is crucial for a wide range of applications, including lab-on-chip devices, biomedical systems, and microreactors.

In traditional macroscopic applications, pressure is measured using mechanical or liquid-based methods; however, their use at the microscale is limited, primarily due to large dead volumes and low spatial resolution. For this reason, current research focuses on miniaturized sensors and integrated measurement systems that enable local and non-invasive pressure measurements directly within the domain of interest.

One of the dominant approaches involves MEMS sensors, particularly piezoresistive and capacitive types. These sensors allow for a high degree of miniaturization and integration into microfluidic systems. For example, Kohl et al. [1] demonstrated an experimental platform with integrated sensors directly within microchannels, enabling in situ pressure measurements during flow. Similarly, Zhang et al. [2] presented an on-chip microfluidic pressure sensor based on the deformation of an elastic structure, capable of real-time pressure monitoring. However, MEMS technologies exhibit certain limitations, such as sensitivity to temperature variations, drift, and limited post-fabrication flexibility.

A significant trend in recent years has been the development of optical pressure sensing methods, which offer high sensitivity and immunity to electromagnetic interference. Optical sensors based on interference, such as Fabry–Perot structures, enable the detection of very small pressure changes with high precision [3]. Another approach involves the use of fiber Bragg gratings, which allow simultaneous measurement of pressure and temperature in small volumes [4]. An overview of these technologies is provided by Lee [5], who highlights their potential for applications requiring high accuracy and long-term stability.

In addition to MEMS and optical methods, microfluidic sensors based on the deformation of compliant materials—typically polymers such as PDMS—are also being developed. These systems exploit changes in channel geometry or fluid interfaces as indicators of pressure. Their advantages include compatibility with microfluidic technologies and relatively simple fabrication; however, their drawbacks include nonlinear response and the need for careful calibration [2].

An alternative approach is represented by indirect pressure measurement methods, which do not require direct sensor integration into the measured domain. Ozsun et al. [6] showed that pressure distribution in a microchannel can be determined based on its deformation.

At the nanoscale, new approaches based on advanced materials such as graphene are emerging. Davidovikj et al. [7] demonstrated a capacitive pressure sensor based on a graphene membrane, capable of detecting extremely small pressure variations at very small sensor dimensions.

Current developments in the field of static pressure sensing in small volumes are characterized by rapid advances in flexible MEMS sensors, optical methods, and the use of nanomaterials. Modern MEMS sensors employ advanced materials and design approaches to enhance sensitivity and mechanical robustness [8,9]. Significant progress has also been achieved in optical methods, where interferometric sensors reach very high resolution and measurement stability [10], with recent review studies confirming the growing importance of these technologies [11].

Another important direction is the use of nanomaterials, particularly graphene and nanostructured layers, which enable the design of extremely sensitive pressure sensors [12,13].

Despite significant progress, several challenges remain in the measurement of static pressure in small volumes. Key issues include achieving high accuracy at very low pressures, minimizing the influence of temperature and material properties, and improving calibration procedures. The issue of measurement accuracy and its impact on applications is also discussed in macroscopic systems, for example in barometric altimetry [14]. The problems of calibration and dynamic response are further addressed by Michael et al. [15].

Current research is therefore directed toward the development of integrated, highly sensitive, and minimally invasive sensors. In our case, it was necessary to measure static pressure under conditions specific to supersonic flow at low pressures.

This article is a continuation in a series reporting on the mapping of physical quantities under low-pressure conditions in supersonic flow within an experimental chamber simulating differential pumping in Environmental Scanning Electron Microscopy (ESEM) [16]. This research is carried out at the Institute of Scientific Instruments of the Czech Academy of Sciences (Brno, Czech Republic) in collaboration with the Department of Electrotechnology at Brno University of Technology (Brno, Czech Republic). ESEM was developed to address challenges associated with the demanding vacuum requirements of conventional electron microscopes. As a result, it enables the observation not only of conductive and semiconductive materials [17,18], but also of non-conductive specimens directly in their native environment without the need for conductive coating [19].

The present work aims to resolve one of the partial tasks within this research, namely, the measurement of static pressure in low-pressure regimes of supersonic flow. This involves experimental measurements under highly specific conditions within a very confined space. Consequently, the methodology requires close integration of experimental sensing using appropriately selected sensors with subsequent comparison of the measured data to CFD analyses performed in Ansys Fluent, supported by theoretical knowledge of the underlying physical phenomena.

The supersonic flow is generated by accelerating gas between two chambers with a large pressure gradient, separated by a small aperture optionally fitted with a nozzle. Due to these large pressure differentials, the flow velocity exceeds 1 Mach, resulting in the formation of shock waves. Such conditions are commonly encountered in ESEM. These shock waves significantly affect the resulting ESEM image, as they induce scattering of the primary electron beam [20,21].

**Figure 1:**
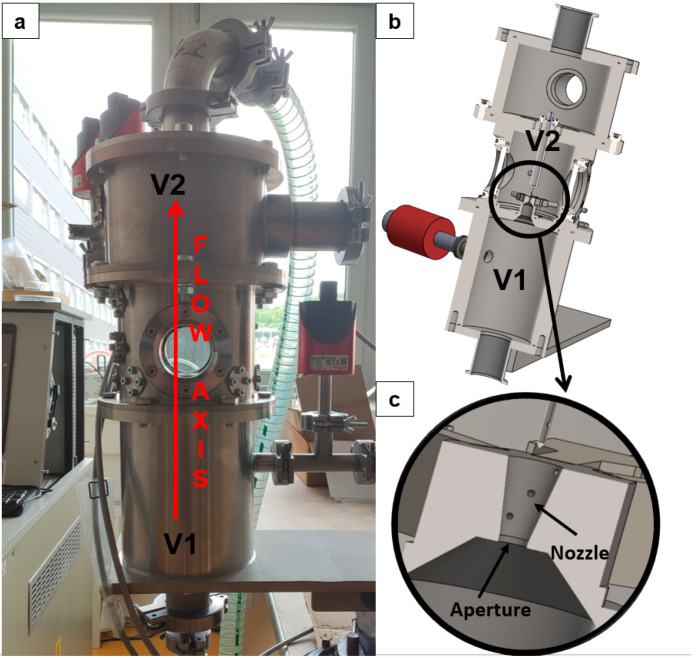
Experimental chamber: real experimental chamber (**a**), 3D solid model (**b**), zoomed area of aperture with nozzle (**c**).

For this reason, an experimental chamber replicating these conditions was constructed (Figure 1a), enabling more detailed investigation of these effects and facilitating mitigation of their impact on electron microscopy. The experimental chamber consists of two chambers, V1 and V2, with a maintained pressure ratio of approximately 10:1 (109,000 Pa in V1 to 9283 Pa in V2). They are separated by an aperture equipped with a nozzle (Figure 1b,c). In addition, this experimental chamber simulates pressures corresponding to altitudes of 16–30 km.

This article focuses on the measurement of static pressure along the flow axis using the Pitot tube method. Due to the small geometric dimensions and low-pressure conditions, as described later, it was not feasible to construct a static pressure sensor with a lateral port positioned according to the standard placement criteria. Therefore, the methodology presented here was employed, in which the experimentally obtained data required subsequent correction, and the final pressure values could be determined only through combined evaluation with CFD analyses. The initial results presented in this work were obtained for pressure ratios between chambers V1 and V2 of approximately 109,000 Pa to 9000 Pa, corresponding to an approximate pressure ratio of 10:1 between the two chambers separated by an aperture equipped with a nozzle.

## 2. Materials and Methods

### 2.1. Pitot Tube for Static Pressure

**Figure 2:**
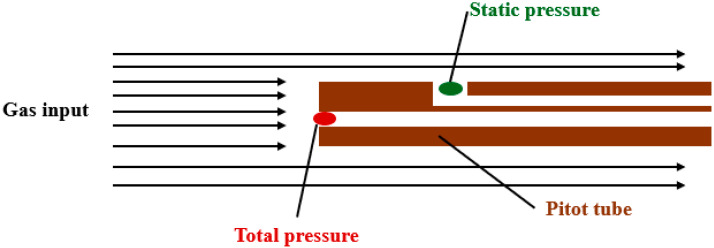
Pitot tube method configuration.

In general, when pressure is measured using Pitot tubes, the probe is inserted into the flow axis and both pressures—static and total—are measured simultaneously. The total pressure is sensed at the probe’s frontal opening, while the static pressure is measured through a lateral port located at a sufficient distance downstream from the probe tip [22] (Figure 2).

**Figure 3:**
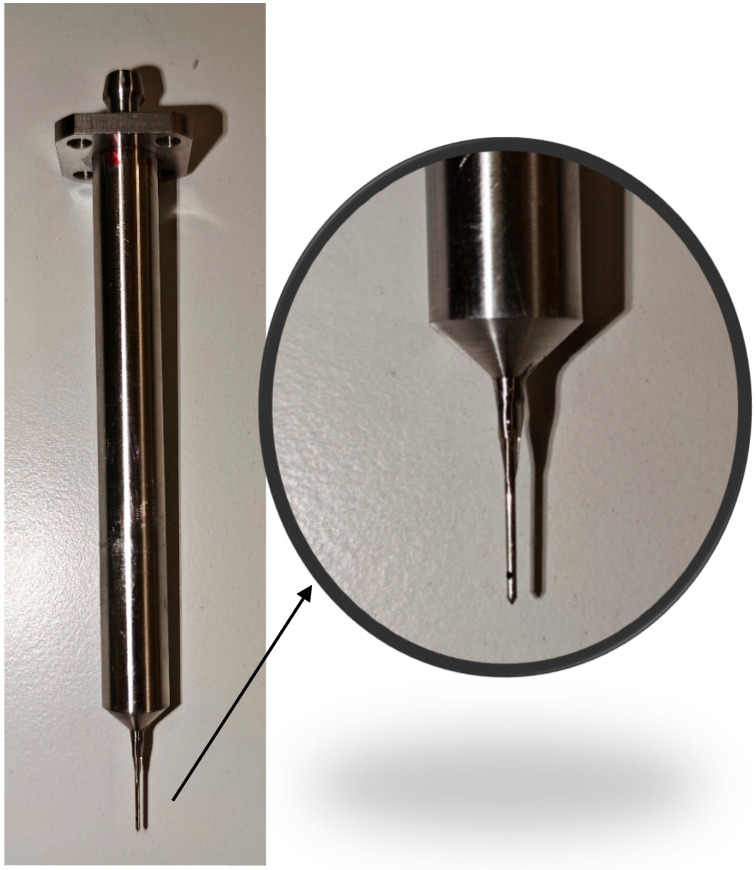
Static pressure tube based on Pitot tube.

However, the experimental chamber described above presents a significant limitation: its dimensions are extremely small compared to those required for conventional Pitot probes [23]. For this reason, total and static pressures were measured separately using pressure sensors operating on the Pitot-tube principle. Static pressure was therefore measured using a sensor equipped with a precisely engineered tip (Figure 3) designed to prevent the formation of a normal shock wave. To achieve the required measurement accuracy, strict tolerance criteria were defined, followed by inspection and fine mechanical adjustment to ensure precise coaxial alignment of the probe with the flow axis [24].

**Figure 4:**
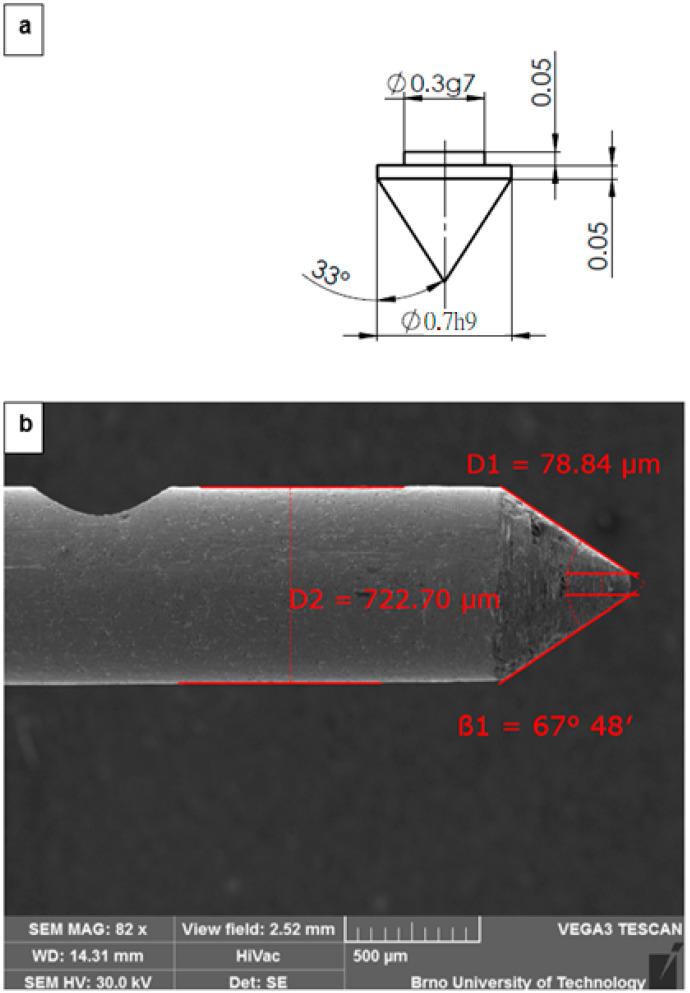
Static pressure sensor based on the Pitot tube principle: diagram of the probe tip with dimensions [mm] (**a**) and actual probe diameters [µm] (**b**).

Subsequently, a post-fabrication inspection of the static-pressure probe was carried out using an electron microscope (Figure 4).

When a Pitot probe is inserted into the flow, the flow field becomes disturbed. For this reason, it is essential to prevent the formation of normal shock wave and instead ensure the formation of an oblique shock. This is achieved through the specific design of the sensor tip, as described above.

In designing the geometry of the Pitot tube for static-pressure measurements, theoretical knowledge of the influence of oblique shock waves on the Mach-number distribution, as well as the relationship between entropy and oblique shock behavior, was applied [25]. An oblique shock wave is generally treated as a discontinuity in flow variables that arises in supersonic flow, for example when the flow interacts with the tip of a Pitot tube. Unlike a normal shock wave, where changes in pressure and velocity occur perpendicular to the flow direction, an oblique shock wave is inclined relative to the flow, making it possible for part of the velocity downstream of the shock to remain supersonic. The transition across an oblique shock is characterized by a sharp increase in pressure, temperature, and density; a reduction in the Mach number in the normal component; and a deflection of the flow direction, while the tangential velocity component remains conserved. Its position and inclination are determined primarily by the upstream Mach number and the flow-deflection angle. With sufficiently large deflection, the shock may transition into a detached shock.

**Figure 5:**
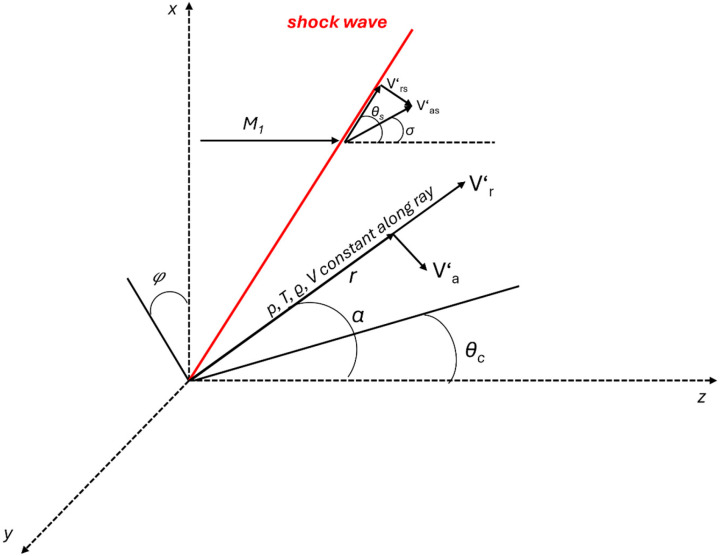
Taylor–McColl theory scheme [26].

Based on Figure 5, a typical three-dimensional conical shock wave can be described. It forms when a uniform supersonic flow with Mach number *M*_1_ impinges on a sharp conical body (e.g., the tip of a Pitot tube), causing the shock to attach directly to the apex of the cone, as described by the Taylor–McColl theory.

The flow around an axisymmetric cone in the supersonic regime generates a spatial conical shock wave that is directly attached to its apex. Assuming steady, axisymmetric, and inviscid flow, the problem can be reduced to the so-called Taylor–Maccoll flow, which describes the radial motion complemented by angularly dependent velocity components. Upstream of the shock, the flow is characterized by a uniform Mach number *M*_1_. Downstream of the shock, the normal component of the velocity decreases significantly, while pressure and temperature increase, and the streamlines are deflected toward the cone by an angle θ.

Across the shock wave itself, the normal Rankine–Hugoniot conditions must be satisfied, determining the post-shock values of the velocity components $V_{r}$ and $V_{\theta }$, as well as the pressure and density immediately downstream. The shock angle β  is the unknown quantity that must be selected such that the numerically integrated solution of the Taylor–Maccoll equation reaches the cone surface smoothly. The resulting flow field exhibits a characteristic conical structure: the streamlines curve toward the cone surface, thermodynamic and flow variables (e.g., pressure or Mach number) depend only on the polar angle, and the entire shock surface forms a conical sheet coaxial with the body.

Correctly determining the distance between the lateral static-pressure tapping point on the probe and its tip is crucial for accurate static-pressure measurement in supersonic flow. Unlike in the subsonic regime, it cannot be assumed that the pressure measured on the probe surface corresponds to the free-flow static pressure. The tapping point must therefore be positioned within the established, undisturbed flow layer. In supersonic flow, this distance typically corresponds to 10–20 probe diameters downstream of the tip [24], which in our case would be 7–14 mm. However, due to the very small dimensions of the experimental chamber, such a placement is not feasible. Maintaining this distance while simultaneously measuring static pressure in the supersonic region near the nozzle exit would require the Pitot-tube tip to extend into the nozzle, which would obstruct the nozzle throat and significantly disturb the flow.

As described above, when the probe is inserted into the supersonic flow, an oblique shock wave forms at its tip. Immediately downstream of this shock, a Prandtl–Meyer expansion occurs, which increases the Mach number back toward the free-flow value, reduces the measured static pressure below the free flow static pressure, and generates a region with a negative pressure coefficient $C_{p}<0$ [27].

The pressure coefficient is defined as

(1) $$C_{p}=\frac{p_{\mathrm{port}}-p_{free}}{q_{free}}$$

where $q_{free}$ is the free-flow dynamic pressure at the measurement location:

(2) $$q_{free}=\frac{1}{2}\rho_{free}U_{free}^{2}$$

where $p_{\mathrm{port}}\;$ is the pressure measured by the probe, and $p_{free}$ is the static pressure at the measurement location without the probe inserted.

From the definition of the pressure coefficient, it follows that $C_{p}=0$ corresponds to an ideal static-pressure measurement, $C_{p}<0$ indicates an expansion region (the measured static pressure will be lower than the free flow static pressure), and $C_{p}>0$ indicates a compression region (the measured static pressure will be higher than the free flow static pressure) [26]. The evaluation of static-pressure tapping based on $C_{p}$ is presented in Table 1. Pressure coefficient $C_{p}$ is defined according to [27]. The classification into ranges (±0.1, ±0.3) is an empirical criterion used to distinguish regions with strong pressure gradients [28,29,30].

As mentioned several times, the small spatial dimensions do not allow significant adjustment of the static-pressure tapping location; therefore, it is necessary to apply a port-interference correction:

(3) $$p_{free}=p_{\mathrm{port}}-C_{p}(x)\;q_{free}$$

Therefore, a methodology was adopted combining experimental measurements with a sensor, where the lateral tapping point, due to the constraints of the small chamber dimensions, was positioned only 2 mm from the probe tip. From a combination of analytical theory for conical flow and CFD analysis, it follows that, for the given probe, the effective tapping location should be approximately 6–9 mm downstream of the tip. A tapping point positioned closer to the tip (e.g., 2 mm, as in this case) inevitably lies within the expansion region and cannot provide an accurate static-pressure measurement without correction.

The experimentally obtained results were compared with those from a calibrated CFD model from Ansys Fluent, in which the inserted probe was explicitly modeled. Excellent agreement was achieved, providing confidence that analogous CFD simulations performed without the probe—i.e., with an unobstructed free flow—yield the desired static-pressure values corresponding to the free-flow conditions along the flow axis. These results were further verified using the aforementioned correction coefficient. The underlying physics is additionally complicated by the necessity to account for boundary-layer effects in the CFD simulations.

In CFD, the values of $C_{p}$ cannot be evaluated directly on the probe wall because the no-slip condition applies at the surface, the pressure is influenced by viscous effects, and the physical static port measures pressure outside the boundary layer. The standard procedure is therefore to sample the pressure along an offset curve located at a distance of several boundary-layer thicknesses from the surface (Equation (1)).

### 2.2. Ansys Fluent Settings

The CFD simulations were performed using Ansys Fluent, a software package designed for solving the Navier–Stokes equations and modeling fluid flow within the framework of continuum mechanics [31,32] as 2D axisymmetric calculation. Additional simulations were performed to compare the axisymmetric 2D approach with a full 3D volumetric model, yielding identical results. There are two main differences, however, which favor the axisymmetric model. The simplifications introduced do not affect the overall outcome but significantly reduce the computational cost. In terms of mesh size, the 2D axisymmetric model for this type of calculation contains just over 200,000 elements, whereas the 3D volumetric model exceeds 6 million elements. Experience shows that although the computational time increases with the number of elements, this dependence is not linear.

The CFD simulations of the flow between two chambers separated by an aperture equipped with a nozzle were carried out in the Ansys Fluent environment. The flow is characterized by a significant pressure drop (approximately 1 atm to 10 kPa) and by the local attainment of supersonic velocities with a Mach number of approximately M≈3 in the region downstream of the nozzle, and was therefore treated as strongly compressible [33]. For this reason, the energy equation was solved, and compressibility effects as well as viscous heating were taken into account.

Viscous heating was taken into account in the simulations by incorporating a viscous dissipation term into the energy equation solved in the Ansys Fluent environment. The calculations were performed using the SST k–ω turbulence model, which enables an accurate description of the flow both in the near-wall region and in areas with strong velocity gradients. By activating the viscous heating option, a source term representing the dissipation of mechanical energy due to viscous effects was included in the energy equation.

The energy equation for compressible flow thus includes the viscous dissipation term *Φ*, which can be expressed in a simplified form as

(4) $$\Phi =\tau_{ij}\frac{\partial u_{i}}{\partial x_{j}}$$

where $\tau_{ij}$ is the viscous stress tensor and $\frac{\partial u_{i}}{\partial x_{j}}$ is the velocity field gradients. This term describes the conversion of the kinetic energy of the flow into internal energy as a result of shear stresses within the fluid. The effect of viscous heating is particularly significant in regions with high shear rates and in supersonic flows. Given the achieved Mach numbers (up to *M* ≈ 3), a non-negligible temperature increase occurs due to energy dissipation, especially in the region downstream of the nozzle and in areas with pronounced velocity and pressure gradients.

This model is therefore suitable for internal flows with strong pressure gradients and potential flow separation. To enhance numerical stability and the physical fidelity of the solution, compressibility corrections were activated within the SST k–ω model. These corrections modify the turbulence production term in regions with high Mach numbers and prevent its overprediction. At the same time, a turbulence production limiter was applied to reduce excessive generation of turbulent kinetic energy in regions with extreme velocity gradients, thereby helping to eliminate non-physical oscillations in the solution and improving the prediction of flow separation. These modifications are particularly important for simulations of compressible and supersonic flows, where the standard formulation of the model may lead to an overestimation of turbulence and subsequent distortion of both the temperature and velocity fields.

A pressure-based coupled solver formulation was selected, as it provides strong coupling between the pressure, velocity, and density fields and exhibits good numerical robustness for complex internal flows with strong gradients and localized shock structures. Although density-based solvers are often used for supersonic compressible flows, test computations in this study showed that they were more sensitive to mesh quality and numerical settings and yielded poorer agreement with the experimental data. In contrast, the pressure-based coupled approach provided more stable convergence and a more accurate prediction of the pressure and velocity fields and was therefore used for all results presented in this work.

The flow variables were discretized using a Second-Order Upwind scheme in order to minimize numerical diffusion and ensure accurate resolution of the strong gradients present in the nozzle region. Gradients were computed using the Least Squares Cell-Based method with Warped Face Gradient Correction enabled to improve accuracy on the unstructured mesh. The numerical results show good agreement with the experimental measurements, confirming the suitability of the chosen numerical configuration for this type of flow.

An adiabatic wall condition was applied at all solid boundaries, corresponding to the high flow velocities and the negligible heat transfer between the flowing medium and the wall [34,35].

The adiabatic boundary condition and viscous heating are not contradictory but describe different physical mechanisms. An adiabatic wall implies zero heat flux across the domain boundary, whereas viscous heating is a volumetric effect included in the energy equation, representing the dissipation of mechanical energy into internal energy. These approaches are therefore consistent: heat generated by viscous dissipation remains within the flowing medium without being transferred through the wall. The use of an adiabatic wall is justified in this case due to the high flow velocities (up to *M* ≈ 3) and the limited time available for heat exchange with the wall, while viscous heating has a significant impact on the resulting temperature field.

The total temperature was set to 297.15 K for all simulated cases.

In the Ansys Fluent setup, the default residual convergence criteria listed in Table 2 were initially used.

In practice, however, the residual thresholds were reduced, as additional iterations were required even after nominal convergence had been achieved, since the monitored quantities had not yet stabilized. The monitored variables included field quantities (spatial values) such as velocity, pressure, and temperature, as well as the pressure at a specific probe location.

Mesh quality was assessed in Ansys Fluent prior to the simulation. The minimum orthogonal quality did not fall below 3 × 10^−1^, and the maximum aspect ratio did not exceed a value of 5.

For the initial simulations, a sensitivity analysis was performed, and adaptive mesh refinement based on the pressure gradient was applied during the computation until the solution no longer indicated the need for further mesh adaptation in regions of shock waves.

In the regions of the aperture, nozzle, Pitot tube, and their surroundings, inflation layers were applied starting directly from the wall [36]. A total of ten layers were specified. The thickness of the first layer was determined using the standard relation for estimating the height of the wall-adjacent cell based on the parameters $y^{+}$, dynamic viscosity μ, friction velocity $U_{\tau }$, and density ρ. The height of the first cell was chosen such that $y^{+}$ did not exceed the value of 1. For the initial setup, this thickness was estimated from anticipated flow parameters and subsequently refined according to the results of the first simulations. The mesh design followed an approach like that used in [37].

### 2.3. Experimental Measurement Settings

**Figure 6:**
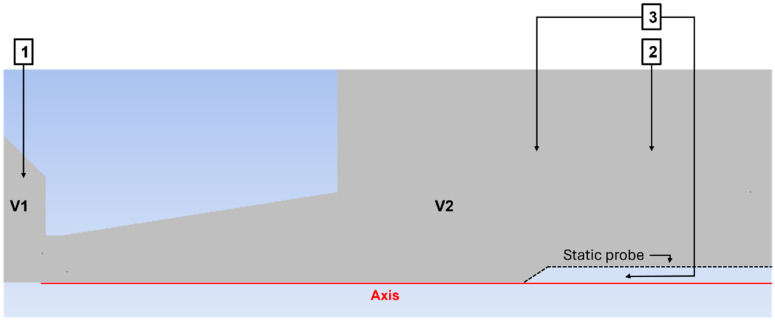
Sensor configuration for total pressure experimental measurements.

Static pressure was measured using the principle of a Pitot tube. Figure 6 shows a two-dimensional axisymmetric schematic of the pressure-sensor arrangement in the experimental setup. Pressure sensors labeled 1 and 2 are absolute pressure sensors [38], while sensor 3 is a differential pressure transducer [39]. Its use was selected to increase measurement accuracy, as a smaller measurement range is associated with lower measurement uncertainty.

The specifications of the individual sensors are provided in Table 3.

Based on the configuration shown in Figure 6, the pressure ratio between chambers V1 and V2 was determined for the specified experimental conditions with a pressure drop of 109,000 Pa to 9000 Pa, using the absolute pressure sensors listed in Table 3. The value of this ratio was approximately 10:1.

A differential pressure sensor [40,41] was integrated into the Pitot tube for static pressure measurement. The data obtained in this manner will subsequently be used for comparison with the results of the CFD simulations.

First, the pressure was experimentally measured at six selected points located at defined distances downstream of the nozzle. These measurements were then compared with CFD analyses that included the simulated inserted probe. The results were evaluated, and subsequently, CFD results were obtained for the same points in the free flow without the inserted probe.

As will become evident from the results, the experimentally recorded pressure corresponds well with the pressure obtained from the CFD analyses with the inserted probe. Based on this calibration, the actual static pressure at the given location in the free flow can be determined. Furthermore, this location is additionally verified using the correction coefficient (Equation (1)).

## 3. Results

**Figure 7:**
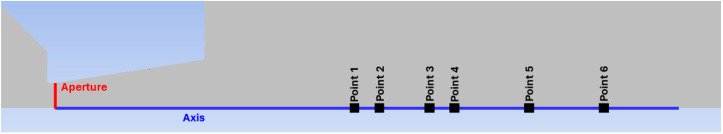
Point positions for experimental measurements with the path (blue line) along which the quantities are plotted.

Table 4 lists the distances of the measured points from the aperture for the static pressure measurements performed using the Pitot tube. Points shown in Figure 7 indicate the positions at which the static pressure is measured through the side port of the Pitot static probe, based on the configuration illustrated in Figure 6, using the sensors listed in Table 3. For static pressure measurement, these points therefore represent the locations where the static pressure was captured; however, the physical tip of the static probe is always positioned 2 mm closer to the aperture. Figure 7 also illustrates the path (blue line) along which the values of the thermodynamic quantities are plotted in the subsequent analyses.

**Figure 8:**
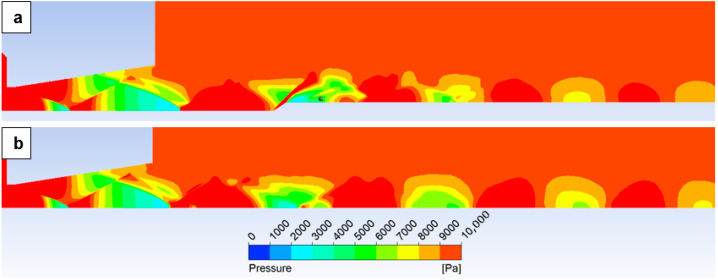
Static pressure distribution for variant with inserted probe at measured point of 13 mm (**a**) and without inserted probe (**b**).

A series of experimental measurements was conducted for the selected points shown in Figure 7. The results obtained using the sensors described above are presented in Table 5 in the *p_experiment_* column. For comparison, the table also includes the pressures extracted from the CFD simulations with the inserted probe in the *p_CFD_* column. For illustration purposes, Figure 8 presents the distribution of static pressure downstream of the nozzle. Figure 8a shows the case with the inserted probe for the variant corresponding to a measurement point located 13 mm downstream. This point, from which the *p_CFD_* value was taken, is marked with a grey dot in Figure 8a. Figure 8b then displays the corresponding static-pressure distribution for the free flow case without the inserted probe.

It is evident that the measurement point lies within the expansion zone; therefore, the measured pressure is lower than the actual pressure that would be present in the absence of the inserted probe. Consequently, the result must be corrected. If it were possible to perform the measurement at a distance of approximately 7 mm downstream of the probe tip, the measurement would already take place in a relaxed region of the typical Mach-diamond pattern. However, as becomes apparent—and as will be demonstrated by the discussion of results in the following section as well as by the findings presented in a subsequent study—at lower ambient pressures, such as those corresponding to altitudes of 15–25 km above sea level, this distance must be increased even further.

Table 5 summarizes the comparison between the experimentally measured pressure values *p_experiment_* obtained using the probe and the pressure values *p_CFD_* determined from the CFD analyses. The agreement is evaluated using the relative error. The error is relatively small and does not exceed the critical threshold of 20% at any point. The table also lists the value of the static pressure in the free flow, $p_{\mathrm{free}}$, obtained from the CFD analyses, which represents the desired quantity. This value can be determined precisely due to the calibrated CFD setup, which was validated by reproducing the pressure values corresponding to the experimentally acquired data using the inserted probe.

A total of eight repeated measurements of the static-pressure differences were performed for the case at a distance of 12 mm for the 109,000 Pa configuration, and the averaged values were subsequently used as the basis for the measurement-error analysis. To assess the measurement accuracy, the standard error of the mean (SEM) was calculated. The SEM, defined as the standard deviation of the sampling distribution of the mean, indicates the expected deviation of the sample mean from the true population mean. The calculation was carried out according to Equation (5), and the corresponding results are summarized in Table 6.

(5) $$SEM=\frac{\sigma }{\sqrt{n}}$$

Based on these eight repeated measurements (Table 6), a detailed uncertainty budget was also constructed in accordance with the ISO/GUM methodology. The budget was computed using Equations (6)–(13), and the resulting values are presented in Table 7.

Measurement model:

(6) $$y=f\left(x_{1},x_{2},\ldots ,x_{N}\right)$$

Sensitivity coefficient:

(7) $$c_{i}={\frac{\partial f}{\partial x_{i}}|}_{x_{1},\ldots ,x_{N}}$$

Covariance between inputs:

(8) $$cov\left(x_{i},x_{j}\right)=r_{ij}\;u\left(x_{i}\right)\;u{(x}_{j})$$

where *r_ij_* is correlation coefficient ($-1\;\leq \;r_{ij}\;\leq 1)$.

Combined standard uncertainty:

(9) $$u_{c}^{2}\left(y\right)=\sum_{i=1}^{N}c_{i}^{2}u^{2}\left(x_{i}\right)+2\sum_{i<j}c_{i}c_{j}\mathrm{cov}\left(x_{i},x_{j}\right)$$

Effective degrees of freedom (Welch–Satterthwaite):

(10) $$\nu_{eff}=\frac{u_{c}^{4}\left(y\right)}{\sum_{i=1}^{N}\frac{c_{i}^{4}u^{4}\left(x_{i}\right)}{\nu_{i}}}$$

Expanded uncertainty:

(11) $$U=k\cdot u_{C}\left(y\right)$$

where *k* is coverage factor z t-distribution:

(12) $$k=t_{1-\alpha ∕2,\nu eff}$$

Measurement result (with 95% confidence interval):

(13) $$y=\hat{y}\pm U$$

In Table 8, verification is performed using the value of $C_{p}$ (the correction coefficient) according to Equation (1). Based on the CFD results of the free flow, the local velocity $v_{\mathrm{free}}$ and density $\rho_{\mathrm{free}}$ were extracted for the evaluated points, and the correction coefficient $C_{p}$ was subsequently determined using Equation (3), from which the corrected pressure $p_{\mathrm{corrected}}$ was obtained.

The table also includes the relative error between $p_{\mathrm{corrected}}$ and the free flow pressure $P_{\mathrm{free}}$. The errors again remain within acceptable limits.

**Figure 9:**
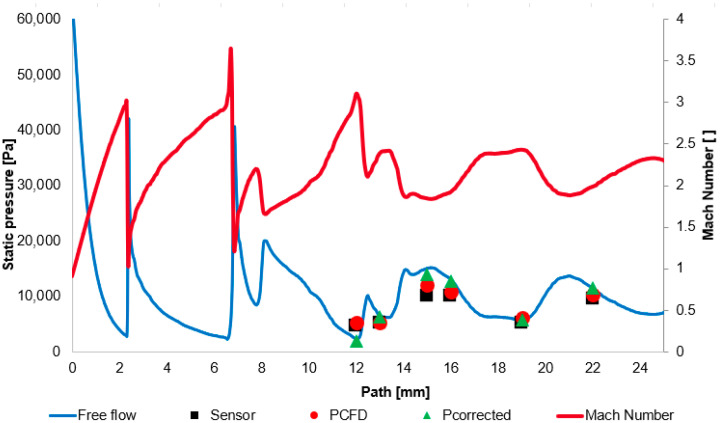
Static pressure and Mach number distribution in free flow.

The corresponding results are presented in the plot in Figure 9. Figure 9 shows the distribution of the Mach number and the static pressure in the free flow. For the evaluated points, a comparison is provided between the experimentally measured values and the CFD results at the point $p_{\mathrm{CFD}}$, followed by the corrected pressure $p_{\mathrm{corrected}}$. The results demonstrate good agreement, particularly for $p_{\mathrm{corrected}}$, which represents the final objective of the applied methodology for determining the true static pressure in the free flow. This value was obtained through the combined use of experimental measurements, CFD analyses, and the relevant theoretical framework.

Given that this study concerns the analysis of an inserted static probe with a tip, it is necessary to assess the influence of the probe’s presence on the investigated flow field. In particular, the character of the shock wave formed on the probe tip must be examined. Table 9 presents the Mach number values for the individual variants and corresponding evaluation points, as obtained from the CFD analyses.

Based on the probe tip angle of 33°, as shown in Figure 4, and the Taylor–Maccoll theory, the corresponding shock wave angle is listed in Table 10. For this cone angle, the boundary Mach number at which the shock detaches from the probe tip is 1.59.

**Figure 10:**
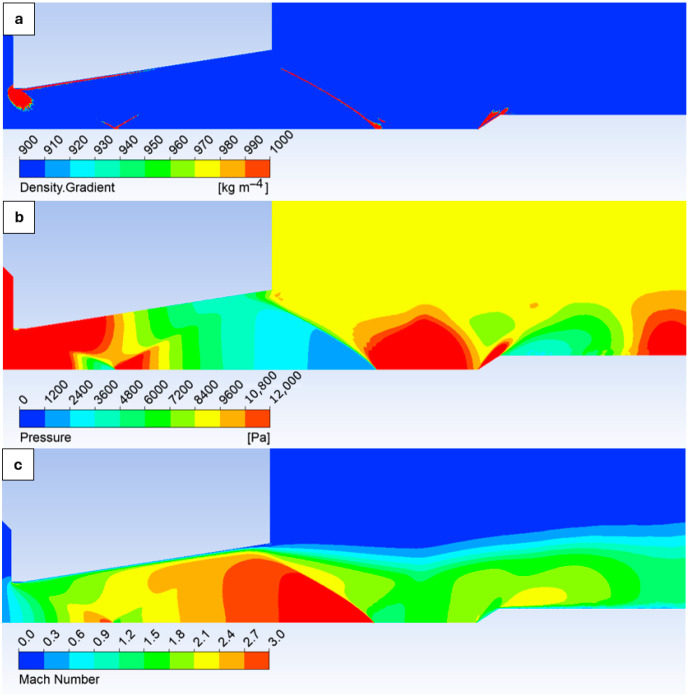
Graphical layout of density gradient (**a**), static pressure, (**b**) and Mach number, (**c**) with the probe inserted at the 19 mm.

The results indicate that, for the probe positioned at 22 mm, the flow conditions already lead to the detachment of the oblique shock wave. For this measurement location, the probe tip angle will therefore need to be adjusted in the next series of experiments. A set of experiments and CFD analyses is likewise planned to determine the influence of the probe tip angle on the possibility of positioning the lateral static port closer to the probe tip. Figure 10 provides a graphical representation of the density gradient (a), static pressure (b), and Mach number (c).

Figure 10 highlights the formation of Mach diamonds, which are intersected by the inserted static probe, and the presence of an oblique shock wave is clearly visible.

## 4. Conclusions

This study addressed the problem of static pressure measurement in low-pressure supersonic flow under severely constrained spatial conditions. Due to extremely small geometric dimensions and low static pressure levels, it was not feasible to design a static-pressure probe with a lateral sensing port positioned at the theoretically recommended location. The primary contribution of this work is therefore the development and validation of a methodology enabling the use of a Pitot probe for static-pressure measurements in supersonic flow even within highly confined geometries, where it is not possible to implement a lateral port at a sufficient distance from the probe tip. The conventional requirement of placing the lateral port at a distance of 10–20 tube diameters was successfully overcome; it was demonstrated that the pressure tapping can be located in the immediate vicinity downstream of the probe cone (static tube), in the present case at approximately two tube diameters, which is approximately 1.4 mm. Consequently, a methodology was established based on a tight integration of experimental measurements—using appropriately selected pressure sensors—and their subsequent comparison with CFD analyses performed in Ansys Fluent, supported by the relevant theoretical framework.

This approach required that the experimentally obtained static-pressure data be corrected using corresponding CFD simulations. A dedicated CFD model was calibrated for the specific flow conditions of the experiment, and the CFD results obtained with the inserted probe were systematically compared with the experimental data. Based on this agreement, a correction coefficient was determined using the free-stream dynamic pressure extracted from the CFD solution. The consistency between experimental measurements and CFD simulations with the probe in place, together with the free-stream CFD analysis and the application of correction-coefficient theory, enabled the determination of the true static pressure in the undisturbed flow, despite the spatial constraints preventing optimal probe design. In the majority of cases, very good agreement between experimental and numerical results was achieved.

The significance of this study extends beyond the measurement methodology itself and finds direct application in EREM research. In such systems, modifications to the geometry and characteristics of the differentially pumped chamber substantially influence the nature of supersonic flow and the formation of shock wave structures, which in turn have a pronounced effect on the dispersion of the primary electron beam. These chambers are characterized by extremely small dimensions, including small apertures and nozzles, where conventional static-pressure measurement techniques are practically infeasible. The proposed methodology thus enables reliable static-pressure measurements under these highly constrained and complex conditions and provides a crucial tool for a deeper understanding of the coupling between flow structures, shock wave behavior, and electron-beam interactions.

Furthermore, the obtained results exhibit broader applicability, particularly in the study of supersonic flows in the stratosphere, where similar spatial and physical constraints are encountered.

Future research will include, among other aspects, an analysis of the influence of probe-tip angle on the magnitude of the required correction, as well as the effect of port location on the spatial extent of the correction region, with the aim of further refining and generalizing the proposed methodology. Upcoming work will also focus on comparing the characteristics of low-pressure supersonic flows with analogous conditions at atmospheric pressure, enabling a clearer definition of the specific features associated with rarefied environments.

An important aspect of future investigations will be the analysis of the influence of experimental geometry, particularly the effect of vacuum chamber dimensions and aperture and nozzle parameters on the structure of the flow field. For all considered configurations, the influence of the lateral sensing-port distance from the probe tip will be examined in relation to the length of the region over which the flow relaxes downstream of the oblique shock wave generated at the probe tip. In this context, particular attention will be paid to the quantitative characterization of the phenomenon whereby this relaxation distance becomes significantly extended under low-pressure conditions compared to standard flow regimes.

## Figures and Tables

**Table 1:** The evaluation of static-pressure tapping based on $C_{p}$.

Range	Description	Suitability
*C_p_* > +0.3	Strong compression	unsuitable
+0.1 < *C_p_* < +0.3	Overpressure region	border
−0.1 ≤ *C_p_* ≤ +0.1	Isostatic region	Correct tapping point
−0.3 < *C_p_* < −0.1	Expansion region	border
*C_p_* < −0.3	Strong expansion	unsuitable

**Table 2:** Residual convergence criteria.

Residual	Absolute Criteria
Continuity	0.001
x–velocity	0.001
y–velocity	0.001
Energy	0.001
k	0.001
omega	0.001

**Table 3:** Specification of each pressure sensor.

-	Sensor Name	Scale	Error
1	Pfeiffer CMR 361 (Pfeiffer Vacuum, Aßlar, Germany)	110 kPa	±0.2% of the measured value
2	Pfeiffer CMR 362	11 kPa	±0.2% of the measured value
3	DPS 300 (BD Sensors, Buchlovice, Czech Republic)	25 kPa	±1% Full Scale Output BFSL (above 0.6 kPa)

**Table 4:** Distances of the measured points from the aperture.

Probe Position	*l* [mm]
Aperture	0
Point 1	12
Point 2	13
Point 3	15
Point 4	16
Point 5	19
Point 6	22

**Table 5:** Results of experimental measuring and CFD analyses.

Variant	*p_experiment_* [Pa]	*p_CFD_* [Pa]	Rel. Error [%]	*p_free_* [Pa]
12	4683	5232	11.7	2300
13	5203	5209	0.12	6620
15	10,112	12,081	19.5	15,139
16	10,134	10,867	7.2	13,190
19	5160	6185	19	5832
22	9471	10,392	9.7	11,607

**Table 6:** Evaluation of the Standard Error of the Mean (SEM) from Measured Pressure Values.

Measurement 1 [Pa]	4683
Measurement 2 [Pa]	4684
Measurement 3 [Pa]	4682
Measurement 4 [Pa]	4683
Measurement 5 [Pa]	4680
Measurement 6 [Pa]	4682
Measurement 7 [Pa]	4685
Measurement 8 [Pa]	4680
Arithmetic mean [Pa]	4682
*σ*	1.77
*SEM* [Pa]	0.625

**Table 7:** Rigorous uncertainty budget following ISO/GUM.

Mean (value of the quantity) $\bar{X}$	4682.4 Pa
Sample standard deviation *s*	1.685 Pa
Standard uncertainty of the mean (Type A) $u_{c}=u\left(\bar{X}\right)=\;\frac{s}{\sqrt{n}}$	0.596 Pa
Degrees of freedom (Welch–Satterthwaite) $\nu_{eff}=n-1$	7
Expansion factor for 95% $k=t_{0.975,7}$	2.365
Expanded uncertainty (95% confidence) $U=k\cdot u_{C}$	1.41 Pa
Final result (95% confidence interval) *y*	4682.4±1.4 Pa

**Table 8:** Verification of results using *C_p_*.

Variant	*C_p_* [-]	*v_free_* [m·s^−1^]	*p_free_* [kg·m^−3^]	*p_corrected_* [Pa]	*p_free_* [Pa]	Rel. Error [%]
12	0.18	636	0.0771	1892	2300	17.8
13	−0.053	573	0.161	6276	6620	5.3
15	−0.085	500	0.289	13,992	15,139	7.6
16	−0.04	512	0.26	12,697	13,190	3.7
19	0.006	578	0.144	5797	5832	0.6
22	−0.01	522	0.233	11,494	11,607	0.9

**Table 9:** Mach number at measurement points according to CFD simulation.

Mach Number [-]						
	12 mm	13 mm	15 mm	16 mm	19 mm	22 mm
109,000 Pa	1.70	2.10	2.18	1.77	1.72	1.56
80,000 Pa	1.74	2.11	2.20	1.80	1.76	1.57
65,000 Pa	1.79	2.15	2.16	1.76	1.78	1.54

**Table 10:** Angle of the shock wave in front of the probe for static pressure measurement.

Shock Wave Angle [°]						
	12 mm	13 mm	15 mm	16 mm	19 mm	22 mm
109,000 Pa	59.7	50.4	49.3	57.2	59.0	detached
80,000 Pa	58.3	50.2	49.0	56.3	57.6	detached
65,000 Pa	56.6	49.7	49.6	57.6	56.9	detached

## Data Availability

The data presented in this study are available on request from the corresponding author.
